# Which types of peripheral nerve blocks should be included in residency training programs?

**DOI:** 10.1186/s12871-015-0001-4

**Published:** 2015-03-12

**Authors:** Marcia A Corvetto, Ghislaine C Echevarria, Ana M Espinoza, Fernando R Altermatt

**Affiliations:** 1 División de Anestesiología, Escuela de Medicina, Pontificia Universidad Católica de Chile, Santiago, Chile; 2 Department of Anesthesiology, New York University School of Medicine, New York, USA; 3 Departmento de Anestesiología, Escuela de Medicina, Universidad de Chile, Santiago, Chile

**Keywords:** Medical education, Peripheral nerve blocks, Survey

## Abstract

**Background:**

Despite the increasing use of regional anesthesia, specific recommendations regarding the type of procedures to be included in residency training programs are not currently available. We aimed to determine the nerve block techniques that practicing Chilean anesthesiologists perceived as essential to master during residency training.

**Methods:**

After institutional ethics committee approval, an online survey was sent to 154 anesthesiologists that graduated between 2005–2012, from the two largest university residency programs in Chile. Multiple-choice questions elicited responses concerning the use of regional anesthesia.

**Results:**

A total of 109 questionnaires were completed, which corresponded to a response rate of 70.8%. Almost all (98.2%) of the respondents used regional anesthesia in their clinical practice, 86.7% regularly performed peripheral nerve blocks (PNBs) and 51% used continuous PNB techniques. Residency programs represented their primary source of training. The most common PNB techniques performed were interscalene (100%), femoral (98%), popliteal sciatic (93%), and Bier block (90%). Respondents indicated that they were most confident performing femoral (98%), Bier block (90%), interscalene (90%), and popliteal sciatic (85%) blocks. The PNBs perceived as essential for their actual clinical practice were femoral (81%), interscalene (80%), popliteal sciatic (76%), and Bier blocks (62%).

**Conclusions:**

Requesting information from former anesthesiology residents may be a source of information, guiding the specific types of PNBs that should be included in residency training. Other groups can easily replicate this methodology to create their own evidence and clinical practice based guidelines for residency training programs.

## Background

Use of peripheral regional anesthesia and analgesia techniques has experienced a significant growth during the last decades [1,2]. Use of these techniques requires procedural competencies that anesthesiologists should acquire as trainees, mostly during the residency program [3,4].

Specific recommendations regarding the type and number of peripheral nerve blocks (PNBs) that should be included in the core curriculum of residency training programs are not currently available [5]. The Accreditation Council for Graduate Medical Education (ACGME) recommends that the minimum clinical experience that should be obtained by each resident consists of “providing clinical care for 40 patients undergoing surgical procedures in whom PNBs are used as part of the anesthetic technique or perioperative analgesic management” [6]. There is a vast spectrum of possible techniques that should be covered during training, so it is unrealistic to attempt to teach all possible techniques before the training period ends.

An alternative approach to determine the types of PNB techniques that must be taught during the residency is to assess the disparities perceived by new anesthesia graduates. They can provide feedback on the specific skills they learned during training and how necessary they were for clinical practice.

The main aim of our study was to determine the types of PNB techniques that Chilean anesthesiologists perceived as essential to be mastered based upon their clinical practice.

## Methods

After the institutional ethics committee (Comité Ético Científico, Escuela de Medicina, Pontificia Universidad Católica de Chile) approved the study, an anonymous web-based survey (SurveyMonkey, www.surveymonkey.com) was sent via email to all anesthesiologists that graduated from one of the two major residency training anesthesia residency programs in Chile between 2005–2012. A total of 154 requests for participation were sent, and a reminder email was sent to each non-responder 2 weeks later. No monetary incentive for survey participation was provided.

The survey consisted of 20 multiple-choice questions that included various aspects of the use of regional anesthesia, with emphasis on PNBs. The survey questions asked for basic demographic information (e.g., age, year since graduation, and type of clinical practice (teaching versus non-teaching)). Other questions were structured to determine the frequency with which PNB and neuraxial anesthesia methods were used, and the clinician’s level of confidence while performing different PNBs. A list of PNBs was provided that included an ordinal scale of “1 = no confidence” to “5 = highly confident”. Anesthesiologists who did not use PNBs were also asked why they did not use these techniques. We also asked respondents to indicate, based upon their clinical practice experience, the PNBs they believed to be essential to master during residency training.

### Statistical analysis

Descriptive statistics were calculated. Chi-square test and Fisher’s exact test were used for inferences on proportions. Subgroup analyses were performed using logistic regression, using the variable PNB (yes/no) as the dependent variable.

Data are expressed as percentage or Odds Ratio (95% confidence interval), unless otherwise stated. A two-sided P value less than 0.05 was considered significant. All analyses were performed using STATA/SE version 12.1 software (StataCorp LP, College Station, TX, USA).

## Results

A total of 109 questionnaires were completed, which corresponded to a response rate of 71%. Most (84%) of the anesthesiologists surveyed were between 30 and 39 years of age. Most of them (46%) were employed at hospitals with 200–400 beds. Forty-four percent of them were involved in teaching and clinical practice.

**Figure 1:**
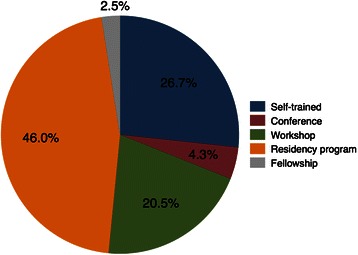
**Possible sources for learning how to perform peripheral nerve blocks.**

Ninety-eight percent of the respondents reported having used regional anesthesia (neuraxial and/or PNBs) in their clinical practice. For two-thirds of them, the use of regional anesthesia corresponded to >30% of their annual clinical practice. Overall, 87% of the anesthesiologists used PNBs regularly, and 51% used continuous PNB techniques. Most of them learned how to perform PNBs during their residency programs (Figure 1).

With respect to peripheral nerve localization techniques, 30% used peripheral nerve stimulation alone, 29% used ultrasound guidance, 41% used a combination of both of these, and none used paresthesia-seeking techniques. Seventy-one percent of the respondents reported that they had ultrasound equipment in their workplaces.

**Figure 2:**
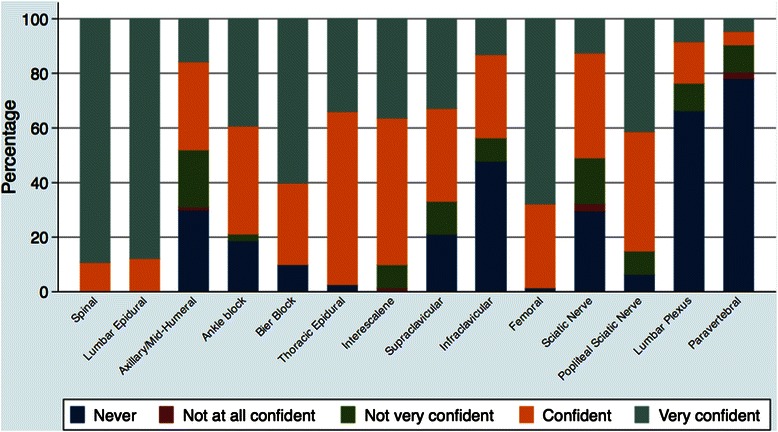
**Degree of confidence in performance of regional anesthesia techniques, ordered by difficulty of the technique.**

**Figure 3:**
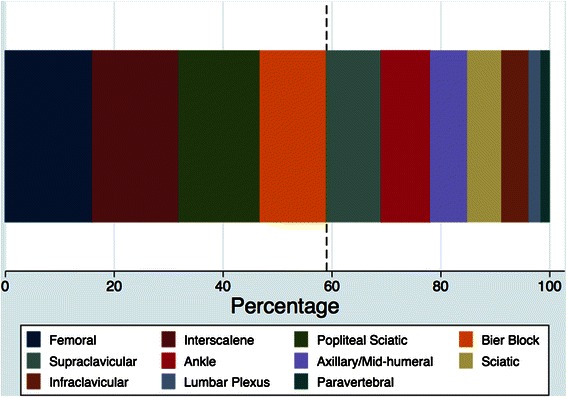
**Peripheral nerve blocks perceived as essential to master to assure optimal performance in the respondents’ current practice.**

Within the group of anesthesiologists that regularly performed PNBs, 100% used interscalene, 99% used femoral, 94% used popliteal sciatic, and 90% used Bier blocks. Figure 2 presents the results for the degree of confidence that each respondent felt when performing the different regional anesthesia techniques, ordered by difficulty [7]. Respondents were most confident (i.e., “very confident” or “confident”) performing spinal anesthesia, epidural techniques (thoracic and lumbar), Bier blocks, and interscalene, femoral, and popliteal sciatic nerve blocks. Finally, regarding blocks perceived as essential to master to ensure optimal performance in their current practice, femoral, interscalene, popliteal sciatic and Bier blocks accounted for a total of almost 60% of the responses (Figure 3).

In the subgroup analysis, the odds of performing PNBs for those respondents working in a teaching hospital/practice was 6.5 times higher than for respondents that worked in a non-teaching practice setting (95% CI: 1.4, 30.6). There were no significant differences in the use of BNPs with respect to the age of the anesthesiologist, years of practice, percentage of cases using regional anesthesia with respect to the annual clinical practice cases eligible to be performed under regional anesthesia, or hospital size (based on number of hospital beds).

## Discussion

Anesthesiologists who graduated from the two largest residency programs (Universidad Católica de Chile, Santiago, and the Universidad de Chile, Santiago) in Chile were surveyed to identify specific peripheral regional anesthesia technical competencies required in clinical practice. The two university-based programs include approximately 70% of the anesthesia graduates in Chile, so the survey population was a representative sample of practicing anesthesiologists. In addition, the survey response rate was >70%. The sample population and response rate assured that our data were valid and were representative of our population of practicing anesthesiologists.

Regional anesthesia represents a significant portion of regular clinical practice among Chilean anesthesiologists. Neuraxial techniques (e.g., spinal and lumbar epidural blocks) are commonly performed in clinical practice with a high level of confidence. This confidence is consistent with the perception that these specific techniques are thoroughly covered by the residency programs. The degree of exposure is great enough to ensure confidence in clinical performance after the training, which far exceeds ACGME requirements. Other studies performed abroad have obtained similar results [8].

A different situation occurs with PNBs. Previous studies in the literature report that a limited number of PNBs are performed during the residency period, and residents lack confidence in their ability to perform these techniques [9]. Specifically, Moon et al. surveyed third clinical anesthesia year residents from 14 residency programs in the United States of America. Authors assessed a pre-specified list of PNBs: interscalene, axillary, femoral, sciatic, popliteal, and lumbar plexus, among the rest of other possible alternatives. According to the authors, these nerve blocks were chosen because they represent frequently- performed blocks in clinical practice.

A wide diversity of described PNB techniques does exist. For instance, the last version of the guidelines for fellowship training in regional anesthesiology and acute pain medicine includes a list of at least twenty-four different types of block techniques that should be known and mastered by regional anesthesia fellows at the end of their training [10]. In the case of anesthesia residency programs, although the ACGME provides specific recommendations in terms of the minimum number of PNBs required at the end of the training period, it does not provide guidelines as to the specific types of blocks. Therefore, it is possible that residents may meet the requirement with a moderate to large number of one or two types of blocks, while being largely unfamiliar and unconfident with many other types.

In order to get better information regarding the actual needs our former residents should face on their clinical practice, our study asked for the techniques perceived as essentials by the anesthesiologist from a broader list of options. A similar methodology was employed by Ouanes et al. [11], but applying the survey among the faculty members of the 26 existing regional anesthesia fellowship programs in the United States and Canada. Based on those survey results, they identified the six anesthesia blocks most often performed at their institutions: interscalene, infraclavicular, supraclavicular, femoral, Labat or subgluteal sciatic, and popliteal sciatic. Although their results have some similarities with ours, the responders in Ounaes’ study were anesthesiologists dedicated to the practice and the teaching of regional anesthesia in academic centers. Our study tries to identify knowledge and skills that every general anesthesiologist should master after a residency program, instead of skills required by a fellow after at the end of at least a one- year training period.

A high proportion of surveyed anesthesiologists performed PNB regularly, and greater than one-half used continuous PNB techniques. There was a concordance between the techniques perceived as essential based upon actual clinical practice, the most commonly used PNBs, and the degree of confidence in performing them. This result may be because the residency programs are thoroughly training the residents in these procedures. Alternatively, alumni who did not acquire the necessary competencies during residency program may have self-trained to achieve the requirements of their clinical duties. The results did not support the second option. The respondents indicated that the primary source of training was the residency programs.

The results indicate that the curricula of Chilean residency programs in anesthesiology should focus on the femoral, interscalene, popliteal sciatic, and Bier nerve blocks. Beyond the general concepts underlying the performance of any PNB in a reliable and safe manner, training should be structured to assure the acquisition of practical skills for optimal performance in clinical practice.

According to the classification and definition of nerve block procedures proposed by Hadzic et al. according to the difficulty of their performance and the time required for mastering them [7], three of these four PNBs are intermediate blocks. The results suggest that the survey participants had reached sufficient expertise to successfully implement them in clinical practice.

A noteworthy aspect of our results was that a high proportion of respondents had ultrasound equipment in the workplace. They regularly used ultrasound as the guidance technique, either alone or in combination with peripheral nerve stimulation. Results previously published by our research team indicate that there has been a rapid increase in the use of ultrasound guided regional anesthesia among Chilean anesthesiologists [12]. The availability of ultrasound technologies experienced a 7-fold growth from 2009–2013. The findings of our current study are also relevant in terms of the contents and the competencies that should be covered by residency curricula. Because ultrasound guided regional anesthesia is often used by our graduates, teaching the fundamentals of ultrasonography, image interpretation, applied sonoanatomy, and teaching specific motor skills (e.g., optimal image acquisition and needle-probe alignment) are critical components that should be included in the residency curriculum.

PNBs were more likely to be used by anesthesiologists in clinical practice associated with a position at an academic institution. This finding was encouraging. The practice of regional anesthesia in academic environments represents a pathway by which the discipline will develop toward consolidation as a subspecialty, and ensure the training of future experts in the field.

There were several limitations of our study. The study sample included participants from only one country, and Chile has a relatively small and homogeneous population of anesthesiologists. The conclusions cannot be extrapolated to other South American countries, but the methodology used can easily be replicated, independent of sample size. Another limitation was that a risk of bias may have been introduced, because the survey was sent by email. Only anesthesiologists who had access to the Internet and used it regularly had the opportunity to respond.

A third study limitation was that the expressions of level of confidence of each anesthesiologist did not necessarily reflect the actual performance (e.g., success rate, rate of complications) of the individual.

## Conclusions

Requesting information from former residents is a valuable source of data, and may be useful for determining the specific types of PNBs that should be included in surgical residency training. The methodology used in this study can be easily replicated to create individual evidence and clinical practice based guidelines for residency training programs worldwide.
